# Identification of Potential Biomarkers for Gut Barrier Failure in Broiler Chickens

**DOI:** 10.3389/fvets.2015.00014

**Published:** 2015-05-26

**Authors:** Juxing Chen, Guillermo Tellez, James D. Richards, Jeffery Escobar

**Affiliations:** ^1^Novus International, Inc., St. Charles, MO, USA; ^2^Department of Poultry Science, University of Arkansas, Fayetteville, AR, USA

**Keywords:** gut barrier function, morphometric analysis, endotoxin, AGP, gene expression, biomarker

## Abstract

The objective of the present study was to identify potential biomarkers for gut barrier failure in chickens. A total of 144 day-of-hatch Ross 308 male broiler chickens were housed in 24 battery cages with six chicks per cage. Cages were randomly assigned to either a control group (CON) or gut barrier failure (GBF) group. During the first 13 days, birds in CON or GBF groups were fed a common corn–soy starter diet. On day 14, CON chickens were switched to a corn grower diet, and GBF chickens were switched to rye–wheat–barley grower diet. In addition, on day 21, GBF chickens were orally challenged with a coccidiosis vaccine. At days 21 and 28, birds were weighed by cage and feed intake was recorded to calculate feed conversion ratio. At day 28, one chicken from each cage was euthanized to collect intestinal samples for morphometric analysis, blood for serum, and intestinal mucosa scrapings for gene expression. Overall performance and feed efficiency was severely affected (*P* < 0.05) by a GBF model when compared with CON group at days 21 and 28. Duodenum of GBF birds had wider villi, longer crypt depth, and higher crypt depth/villi height ratio than CON birds. Similarly, GBF birds had longer crypt depth in jejunum and ileum when compared with CON birds. Protein levels of endotoxin and α1-acid glycoprotein (AGP) in serum, as well as mRNA levels of interleukin (IL)-8, IL-1β, transforming growth factor (TGF)-β4, and fatty acid-binding protein (FABP) 6 were increased (*P* < 0.05) in GBF birds compared to CON birds; however, mRNA levels of FABP2, occludin, and mucin 2 (MUC2) were reduced by 34% (*P* < 0.05), 24% (*P* = 0.107), and 29% (*P* = 0.088), respectively, in GBF birds compared to CON birds. The results from the present study suggest that serum endotoxin and AGP, as well as, gene expression of FABP2, FABP6, IL-8, IL-1β, TGF-β4, occludin, and MUC2 in mucosa may work as potential biomarkers for gut barrier health in chickens.

## Introduction

Barrier function is a critical aspect of gut heath. Oxidative stress, poorly digestible protein, and coccidiosis are some examples that can cause gut barrier failure (1–5). Nevertheless, as a consequence of the removal of anti-microbial growth promoters, new multifactorial diseases causing enteritis and gut disorders of unknown origin have emerged in broilers, causing negative impacts in health and performance (6–9). Among them, dysbacteriosis, defined as the presence of a qualitatively and/or quantitatively abnormal microbiota in the proximal parts of the small intestine, associated with reduced nutrient digestibility, impaired intestinal barrier function, bacterial translocation, and inflammatory responses have been reported (4, 5, 10). However, more recently, poor gut health has also been associated with bacterial chondronecrosis with osteomyelitis in broiler chickens and breeders (11–13). As the largest organ in the body, the gut serves as a selective barrier to take up nutrients and fluids into the body, while excluding undesirable molecules and pathogens (3, 14, 15). Therefore, proper gut barrier function is essential to maintain optimal health and balance throughout the body, and represents a key line of defense against foreign antigens from the environment (16). The first layer of gut barrier is the extrinsic mucus layer comprised an outer layer associated with bacteria and an inner layer with high concentrations of secretory IgA and mucin. The outer layer is loosely attached to epithelium. The inner layer is adherent to the second layer of gut barrier, the intestinal epithelial cells (IEC). IEC are a single layer of epithelial cells that separate the intestinal lumen from underlying lamina propria (17–19). These epithelial cells must be able to rapidly regenerate in the event of tissue damage (14, 20, 21). The enterocytes in the apical epithelium are responsible for absorption of nutrients. Tight junctions (TJ) seal the paracellular space between adjacent epithelial cells and regulate the permeability of intestinal barrier by preventing diffusion of microorganisms and antigens (22, 23). Since IEC are the primary cell type coming into contact with the external environment, they act as the host’s first line of the defense. In spite of their non-hematopoietic derivation, IEC represent a core element of innate immunity within the gut-associated lymphoid tissue, displaying a wide array of immune functions. In fact, IEC are able to recognize pathogens through the expression of innate immune receptors, release of anti-microbial molecules, and secretion of a wide number of hormones, neuro transmitters, enzymes, as well as cytokines and chemokines that link innate and adaptive immune responses (24–26). Hence, any direct or indirect damage on IEC may cause a breakdown in gut barrier and consequently, disruption of normal mucosal immune homeostasis that can potentially lead to uncontrolled chronic intestinal and systemic inflammation (27, 28).

Several investigators have described the pathways associated with the disruption of the protein networks that connect epithelial cells by inflammatory mediators, such as hormones, oxygen free radical species, enzymes, as well as multiple proinflammatory cytokines (27, 29, 30). Feeding oxidized/unpreserved fat has been also shown to increase intestinal epithelial turnover rates and increase apoptosis at villus tips in poultry and swine (31). Non-starch polysaccharides (NSP), such as β-glucans and pentosans have been shown to have a detrimental influence on the utilization of nutrients in broilers by increasing digesta viscosity and reducing digestibility of nutrients (e.g., fat and protein) (32, 33), which could cause dysbacteriosis. Currently, no biomarkers have been described as tools to evaluate gut inflammation or gut barrier failure in broiler chickens. The objective of the present study was not to determine the individual effects of diet ingredients or coccidia challenge on gut health, rather to identify potential biomarkers for gut barrier failure. Therefore, we attempted to exacerbate gut barrier failure by feeding a high NSP diet containing rye, wheat, and barley to induce high digesta viscosity (4, 5) in combination with a 2× coccidiosis vaccination to induce gut health challenge.

## Materials and Methods

### Animal source and diets

A total of 144 day-of-hatch Ross 308 male broiler chickens were randomly housed in 24 battery cages with six chicks per cage in environmentally controlled rooms. To avoid cross contamination of coccidiosis vaccine, birds in control group (CON) and gut barrier failure (GBF) group were housed in two separate but identically controlled rooms. Temperature was maintained at 34°C for the first 5 days and then gradually reduced according to normal management practices, until a temperature of 23°C was achieved. Lighting was provided for 24 h/day. During the first 13 days, birds in CON or GBF groups were fed common corn–soy starter diet (Table 1). On day 14, birds in CON group were switched to a corn–soy grower diet (14–28 days) and the GBF group was switched to rye–wheat–barley diet (Table 1). The experimental diets were formulated to approximate the nutritional requirements of broiler chickens (34). On day 21, birds in GBF treatment were orally challenged with 2× dose of Advent™ coccidiosis vaccine, a mixture of *Eimeria acervulina*, *Eimeria maxima*, and *Eimeria tenella* (Huvepharma Sofia, Bulgaria). All research procedures were reviewed and approved by a licensed veterinarian and also followed the protocols described previously (5, 35), which were approved by IACUC at University of Arkansas. All studies performed by Novus International, Inc. are in accordance to the standards of the Guide for the Care and Use of Agricultural Animals in Research and Teaching (35).

**Table 1:** **Ingredient composition and nutrient content of common, control, and gut barrier failure (GBF) diets, as-is basis**.

Ingredient	Common starter, 0–13 days (%)	Control grower, 14–28 days (%)	GBF grower, 14–28 days (%)
Corn	60.6	60.6	0
Rye	0	0	33.95
Wheat	0	0	20
Barley	0	0	10
SBM, 47.5% CP	32.56	32.56	28.8
Soybean oil	1.08	1.08	2.96
L-lysine HCl	1.48	1.48	0.14
MHA^®^	0.3	0.3	0.42
L-threonine	0.01	0.01	0.05
L-tryptophan	0.14	0.14	0.11
Dicalcium phosphate, 18.5%	1.59	1.59	1.57
Limestone	1.09	1.09	1
Salt	0.25	0.25	0.25
Choline chloride, 60%	0.25	0.25	0.25
Sodium bicarbonate	0.2	0.2	0.2
Mineral premix^a^	0.2	0.2	0.2
Vitamin premix^b^	0.1	0.1	0.1
Santoquin™ Mixture 6	0.02	0.02	0
MycoCURB™	0.05	0.05	0
Coban^®^ 90	0.05	0.05	0
BMD^®^ 60	0.03	0.03	0
**Calculated nutrients**
ME, kcal/kg	3,031	3,152	3,152
SID Lysine, %	1.27	1.1	1.1
SID TSAA, %	0.94	0.84	0.84
Total CP, %	22	20.7	21.8
Ca, %	1.05	0.9	0.9
Available P, %	0.5	0.45	0.45

*^a^Mineral premix supplied per kilogram of diet: Mn, 120 mg; Zn, 100 mg; Fe, 40 mg; Cu, 16 mg; I, 1.25 mg; Se, 0.30 mg*.

*^b^Vitamin premix supplied per kilogram of diet: retinol, 9.2 mg; cholecalciferol, 100 μg; dl-α-tocopherol, 90 mg; menadione, 6 mg; thiamine, 6.2 mg; riboflavin, 26.5 mg; pantothenic acid, 39.7 mg; niacin, 100 mg; pyridoxine, 11 mg; folic acid, 4 mg; biotin, 0.3 mg; cyanocobalamin, 0.1 mg*.

### Experimental design

The 144 day-of-hatch chickens were randomly allotted to one of two groups; CON or GBF on the basis of initial body weight (BW). Each treatment was comprised of 12 replicates of six chicks each (*n* = 72/group). At 21 and 28 days, BW, body weight gain (BWG), and feed intake (FI) were recorded in each cage to calculate feed conversion ratio (FCR).

### Sample collection

At 28 days of age, one chicken from each cage was euthanized by CO_2_ asphyxiation for sample collection. Blood sample was taken from cardiac puncture using a syringe, kept at room temperature for 3 h to allow clotting, and centrifuged (1,000 × *g* for 15 min at 4°C) to separate serum. Following euthanasia, a 1-cm section of duodenum was collected from the middle of the descending duodenum; a 1-cm section of jejunum was collected at the Meckel’s diverticulum; a 1-cm section of ileum was collected 2 cm before the ceca. All of intestinal sections were rinsed with 10% neutral buffered formalin and then fixed in 20× volume of 10% neutral buffered formalin. A 10-cm section of jejunum was rinsed with ice cold phosphate buffered saline (pH 7.4) and cut open to scrape mucosa using RNAse-free glass slides into 2-ml tubes with 1 ml RNAlater (Applied Biosystems, NY, USA). The mucosal scrapings were stored at 4°C for 24 h and then at −20°C until total RNA isolation.

### Histological sample preparation and intestinal morphometry measurement

Intestinal segments were trimmed, processed, and embedded in paraffin. A 5-μm section of each sample was placed on a glass slide and stained with hematoxylin and eosin for morphometry examination and measurement under Olympus light microscope using Olympus MicroSuite™ Imaging software (Center Valley, PA, USA). Five replicate measurements for each variable studied were taken from each sample, and the average values were used in statistical analyses. Villi height was measured from the top of the villi to the top of the submucosa. Crypt depth was measured from the base upwards to the region of transition between the crypt and villi. Villi width was measured at the middle of each villus, whereas crypt/villi ratio was determined as the ratio of crypt depth to villi height (36).

### Serum endotoxin and serum α1 acute phase protein determination

Endotoxin was measured using a chicken Endotoxin Elisa kit from Amsbio (Cambridge, MA, USA). Acute phase protein, α1-acid glycoprotein (AGP) was measured using chicken α1-acid glycoprotein measurement kit from The Institute for Metabolic Ecosystem (Miyagi, Japan). The Optical Density for both kits was determined at 450 nm using a BIO-TEK ELx800 (BIO-TEK Instrument, Winooski, VT, USA).

### Quantitative reverse transcription polymerase chain reaction (qRT–PCR)

Total RNA was isolated from mucosa scraping samples using Clontech Total RNA isolation NucleoSpin^®^ RNA II kit (Clontech Laboratories, Inc., CA, USA). One microgram of total RNA, 11mer oligo mix from Fluoresentric, and M-MLV Reverse Transcriptase (Life Technologies, Grand Island, NY, USA) were used to synthesize cDNA according to the manufacturers’ instructions. The relative mRNA levels of mucin 2 (MUC2), fatty acid-binding protein (FABP) 2, FABP6, interleukin (IL)-8, IL-1β, transforming growth factor (TGF)-β4, occludin, zonula occluden (ZO)-1, junctional adhesion molecule (JAM) 2, JAM3, catenin, tumor necrosis factor (TNF) α, Toll-like receptor (TLR) 2β, TLR4, and claudin 1 were measured by quantitative PCR using Applied Biosystems^®^ SYBR^®^ Green PCR Master Mix, the 7500 Fast Real-Time PCR System, and primers in Table 2. Results were expressed as the level relative to the corresponding housekeeping gene *actin*. All primers were verified for the efficiency and linearity of amplification.

**Table 2:** **List of primers used for qRT–PCR**.

Genes	Forward primer	Reverse primer	Fragment size (bp)
Actin	CAACACAGTGCTGTCTGGTGGTA	ATCGTACTCCTGCTTGCTGATCC	205
MUC2	GCCTGCCCAGGAAATCAAG	CGACAAGTTTGCTGGCACAT	59
FABP2	AAAGATAATGGAAAAGTACTCACAGCAT	CCTTCGTACACGTAGGTCTGTATGA	77
FABP6	CGGTCTCCCTGCTGACAAGA	CCACCTCGGTGACTATTTTGC	59
IL-8	TCCTGGTTTCAGCTGCTCTGT	CGCAGCTCATTCCCCATCT	52
TGF-β4	CGGCCGACGATGAGTGGCTC	CGGGGCCCATCTCACAGGGA	113
Occludin	GAGCCCAGACTACCAAAGCAA	GCTTGATGTGGAAGAGCTTGTTG	68
ZO1	CCGCAGTCGTTCACGATCT	GGAGAATGTCTGGAATGGTCTGA	63
JAM2	AGCCTCAAATGGGATTGGATT	CATCAACTTGCATTCGCTTCA	59
JAM3	CCGACGGCTGTTTGTGTTT	GGCGGTGCAAAGTTTTGG	56
Catenin	CGACAACTGCTCCCTCTTTGA	GCGTTGTGTCCACATCTTCCT	63
TNFα	TGTTCTATGACCGCCCAGTTC	GACGTGTCACGATCATCTGGTT	63
TLR2β	CGCTTAGGAGAGACAATCTGTGAA	GCCTGTTTTAGGGATTTCAGAGAATTT	90
TLR4	AGTCTGAAATTGCTGAGCTCAAAT	GCGACGTTAAGCCATGGAAG	190
Claudin 1	TGGCCACGTCATGGTATGG	AACGGGTGTGAAAGGGTCATAG	62
IL-4	GCCAGCACTGCCACAAGA	GGAGCTGACGCGTGTTGAG	54
IL-6	GAGGGCCGTTCGCTATTTG	ATTGTGCCCGAACTAAAACATTC	67
IL-1β	CAGCCCGTGGGCATCA	CTTAGCTTGTAGGTGGCGATGTT	59

### Statistical analyses

All data were tested for normality and subjected to one-way ANOVA as a completely randomized design using the GLM procedure of SAS (37). Each cage was used as the experimental unit for the analysis. Growth performance including BW, BWG, FI, and FCR used the average data per cage. Gut morphometric measurements, serum endotoxin, AGP, and qRT–PCR used individual measurement from one randomly chosen bird per cage. Data are expressed as mean ± SE.

## Results

### Growth performance

The results of the growth performance parameters between CON and GBF groups are summarized in Table 3. BW, FI per bird, BWG and FCR at 21 and 28 days of age were dramatically reduced in GBF chickens when compared with CON chickens (*P* < 0.05), indicating that GBF model substantially compromised the growth performance of chickens.

**Table 3:** **Performance parameters between control and gut barrier failure grower groups (GBF)**.

Treatments and growing phase	BW (g)	FI per bird (g)	BWG (g)	FCR during each phase
**21 days**
CON	866.25 ± 11.87^a^	617.25 ± 8.76^a^	390. 58 ± 6.21^a^	1.58 ± 0.02^b^
GBF	642.58 ± 10.50^b^	480.67 ± 11.85^b^	203.17 ± 11.27^b^	2.42 ± 0.09^a^
**28 days**
CON	1,302.75 ± 26.45^a^	729.50 ± 26.17^a^	436.50 ± 19.04^a^	1.67 ± 0.03^b^
GBF	895.50 ± 21.58^b^	578.17 ± 9.5^b^	252.92 ± 20.30^b^	2.42 ± 0.17^a^

*Data are expressed as mean ± SE*.

*^a,b^Superscripts within columns indicate difference at *P* < 0.05*.

### Histomorphometric analysis

**Figure 1:**
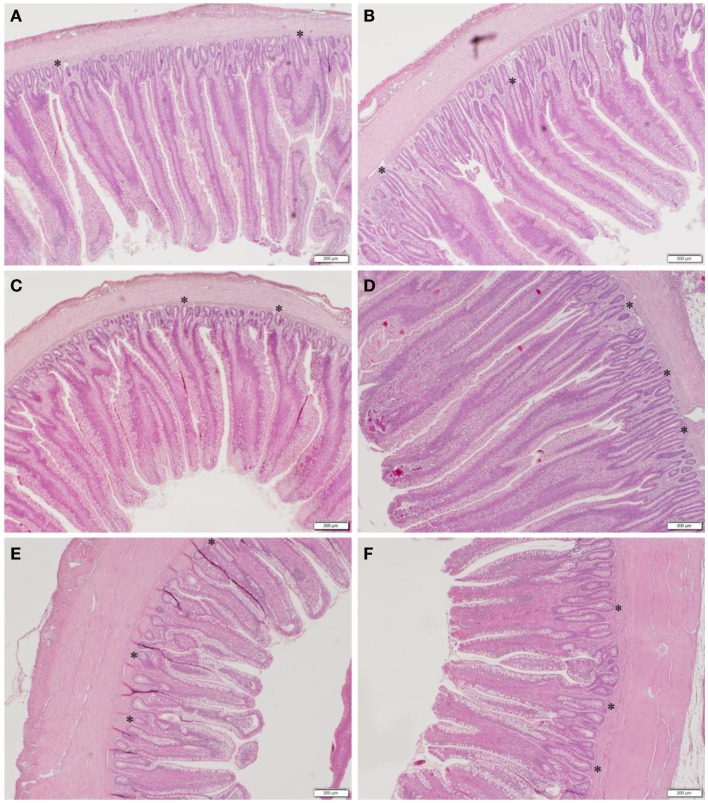
**Representative images of duodenum (A,B), jejunum (C,D), and ileum (E,F) in control (A,C,E) and gut barrier failure (B,D,F) groups of broilers chickens at 28 days of age**. The representative crypts are shown as *.

The results of the histomorphometric analysis of duodenum, jejunum, and ileal tissue between CON and GBF chickens at 28 days of age are summarized in Table 4. The duodenum, jejunum, and ileum all showed increased (*P* < 0.05) crypt depth (shown as * in Figure 1) in GBF chickens compared to CON chickens. GBF chickens also had wider villi in duodenum and jejunum, and higher crypt/villi ratio in duodenum compared to CON chickens; however, the crypt/villi ratio was not different in jejunum (*P* = 0.064) and ileum (*P* = 0.208) because the villus height in jejunum and ileum was also increased (*P* < 0.03) in GBF birds compared to CON birds. The increase of crypt depth and/or the crypt/villi ratio is an indication of greater need of cell proliferation to maintain proper gut health, which suggests that GBF model generated unhealthy gut barrier.

**Table 4:** **Histomorphometric analysis of duodenum, jejunum, and ileum in control (CON) and gut barrier failure (GBF) groups in chickens at 28 days of age**.

Tissue	CON	GBF
**Duodenum**
Villus height, μm	2324.7 ± 123.84^a^	2649.8 ± 156.21^a^
Villus width, μm	172.81 ± 5.24^b^	214.08 ± 13.04^a^
Crypt depth, μm	104.51 ± 4.76^b^	201.74 ± 17.10^a^
Crypt/villi ratio	0.04 ± 0.01^b^	0.08 ± 0.01^a^
**Jejunum**
Villus height, μm	1883.40 ± 141.54^b^	2273.80 ± 77.17^a^
Villus width, μm	170.57 ± 9.17^b^	190.02 ± 12.08^a^
Crypt depth, μm	112.84 ± 9.32^b^	172.78 ± 10.59^a^
Crypt/villi ratio	0.06 ± 0.01^a^	0.07 ± 0.01^a^
**Ileum**
Villus height, μm	1005.70 ± 45.77^b^	1334.13 ± 79.61^a^
Villus width, μm	163.80 ± 4.97^a^	166.25 ± 7.85^a^
Crypt depth, μm	113.63 ± 7.91^b^	174.70 ± 14.11^a^
Crypt/villi ratio	0.11 ± 0.01^a^	0.13 ± 0.01^a^

*Values are expressed as means ± SE*.

*^a,b^Superscripts within rows indicate difference at *P* < 0.05*.

### Serum endotoxin and AGP

Table 5 shows the comparison of serum AGP and endotoxin levels between CON and GBF groups of broiler chickens at 28 days of age. AGP, a marker for systemic inflammation, was increased (*P* < 0.05) by 3.8-fold in GBF birds compared to CON birds (Table 5), suggesting that systemic inflammation was occurring in GBF birds. Endotoxin, a toxin released by gram-negative bacteria in the gut, was increased (*P* < 0.05) by 2.1-fold in serum of GBF birds compared to CON birds (Table 5), which suggests that greater amount of endotoxin was translocated from intestinal lumen into blood.

**Table 5:** **Comparison of serum endotoxin and α1 acute phase protein (AGP) values between control and gut barrier failure groups in chickens at 28 days of age**.

Treatment	Endotoxin pg/ml	α1 Acute phase protein (AGP) μg/ml
CON	159.03 ± 8.56^b^	174.40 ± 28.95^b^
GBF	331.84 ± 80.46^a^	655.30 ± 6.38^a^

*Data are expressed as mean ± SE*.

*^a,b^Superscripts within columns indicate difference at *P* < 0.05*.

### Gene expression in jejunal mucosa by qRT–PCR

The relative mRNA levels of genes that are possibly involved in gut barrier function and inflammation in jejunal mucosa of broilers chickens at 28 days of age are shown in Table 6. The relative mRNA levels of IL-8, IL-1β, TGF-β4, and FABP6 were increased (*P* < 0.04) by 3-, 1.5-, 2.2-, and 7-fold, respectively, in GBF chickens compared to CON chickens. However, FABP2, occludin, and MUC2 mRNA levels were decreased by 34% (*P* = 0.005), 24% (*P* = 0.107), and 29% (*P* = 0.088), respectively, in GBF birds compared to CON birds. The mRNA levels of catenin, claudin 1, ZO1, JAM2, JAM3, IL-4, IL-6, TLR4, TLR2β, and TNF-α were not different (*P* > 0.1) between CON and GBF chickens (data not shown).

**Table 6:** **Relative mRNA levels of genes in jejunal mucosa between control and gut barrier failure groups of broilers chickens at 28 days of age**.

Treatment	mRNA TGF-β4	mRNA IL-1β	mRNA IL-8	mRNA FABP2	mRNA FABP6	mRNA MUC2	mRNA occludin
CON	81.98 ± 4.55^a^	25.6 ± 4.52^a^	0.04 ± 0.01^a^	26.72 ± 1.99^b^	0.01 ± 0.001^a^	123.30 ± 15.51^b^	2.23 ± 0.26^b^
GBF	182.03 ± 18.09^b^	43.89 ± 6.65^b^	0.13 ± 0.01^b^	17.66 ± 1.89^a^	0.07 ± 0.01^b^	87.11 ± 12.16^b^	1.69 ± 0.16^b^
*P*-value	0.0001	0.040	<0.0001	0.0005	0.020	0.088	0.107

*Data are normalized by actin mRNA and expressed as mean ± SE*.

*^a,b^Superscripts within columns indicate difference at *P* < 0.05*.

## Discussion

It is well known that poor gut health causes negative impacts in the health and growth performance of broiler chickens in poultry industry. Alternative grains, such as wheat, barley, and rye that are high in NSP, have been reported to cause a significant reduction in performance (38–40). Several mechanisms of the action of NSP on nutrient absorption have been described including an increased digesta viscosity due to reduced digestibility, thickening of the mucous layer on the intestinal mucosa, epithelial cell apoptosis, and inflammation caused by dysbacteriosis (10, 31, 39). Poultry have little or no intrinsic enzymes capable of hydrolyzing these NSP, so high concentrations of NSP in wheat, barley, or rye lead to reduced nutrient digestibility. The undigested feed ingredients in the gut provide nutrients for bacteria overgrowth in the hind gut, leading to dysbacteriosis. High NSP diets have also been associated with necrotic enteritis, a multifactorial disease caused by *Clostridium perfringens* that is probably the most important bacterial disease in terms of economic implications in broiler chickens (41). The nutritional and economic consequences of mounting an inflammatory response in poultry are inversely related to BWG and overall performance (42, 43). In the present study, a wheat–barley–rye diet in combination with a coccidia challenge was used to induce gut barrier failure in broiler chickens. The overall growth performance and feed efficiency were severely reduced by this GBF model. These results are in agreement with previous studies that high NSP diets compromised growth performance in chickens (4, 5, 44, 45).

The morphometry of duodenum, jejunum, and ileum in CON and GBF chickens at 28 days of age was measured under microscope to confirm whether the rye–barley–wheat diet and coccidia challenge generated gut barrier failure. GBF birds had longer crypt depth than CON birds in duodenum, jejunum, and ileum and also higher crypt/villi height ratio in duodenum than CON birds. Crypt depth and the ratio of crypt depth to villus height are measures of efficiency because the increase of crypt depth and/or crypt/villi ratio indicates greater need of cell proliferation to maintain gut barrier integrity (46–48). In addition to longer crypt depth, duodenum and jejunum of GBF birds also had wider villi. Narrow villi have greater nutrient absorption area. Widening of villus indicates less nutrient absorption area and probably also greater amount of gut-associated immune tissue proliferation and accumulation in the villus, which is another indication of compromised gut heath. The structural change in GBF birds confirmed that gut barrier failure was occurring in GBF birds, which may be associated with the poor performance in this study and is consistent with a previous study (49).

The gastrointestinal tract (GIT) is repeatedly challenged by foreign antigens and the intestinal mucosa must have the capability of fast restoration in the event of tissue damage (50). Impairment of this fragile barrier leads to enteritis and other inflammatory diseases (9). The intestinal mucosa contains different types of epithelial cells with specific functions. IEC control surface-associated bacterial populations without upsetting the microbiome that are vital for host health (51), and play an essential role in maintaining gut homeostasis and barrier function (52, 53). As a single-cell layer, IEC serve as a protective barrier against the external environment and maintain a defense against intraluminal toxins and antigens in addition to support nutrients and water transport (54). IEC are sealed together by adherens junctions (AJ) and TJ that are composed of cadherins, claudins, occludins, and JAM (29, 55–57). Upon injury, IEC undergo a wound healing process that is reliant on three cellular events: restitution, proliferation, and differentiation (27). Previous studies have shown that various regulatory peptides, including growth factors and cytokines, are capable of influencing the restoration of damaged IEC (58).

Gram-negative bacteria in the gut release endotoxin during growth, division, and death, and luminal endotoxin can translocate to circulation via two routes: (1) non-specific paracellular transport through TJ of epithelial cells, and (2) transcellular transport through lipid raft membrane domains and receptor-mediated endocytosis (2, 59). TLR4 is involved in the latter route (60). The lack of difference of TLR4 mRNA levels between CON and GBF birds suggests that endotoxin probably did not enter into circulation via transcellular transport. Pathogens, such as *Escherichia coli* or *C. perfringens*, as well as their elaborated toxins (e.g., endotoxin or entertoxin) have been reported to alter epithelial TJ and gut barrier function (23). Poor integrity of gut barrier or opening of TJ has been reported to facilitate paracellular transport of endotoxin, which will increase proinflammatory cytokine secretion and activate innate and adaptive immune response (61, 62). Secreted cytokines may enter the IEC through the basolateral side, resulting in further increased inflammation, disruption of TJ complexes, and increased paracellular endotoxin transport (63). Interestingly, there were detectable levels of endotoxin in CON chickens, which are actually not the background noise detected by ELISA kit. In this study, the CON chickens were much healthier than GBF chickens, the endotoxin in the serum of CON chickens could be non-specific paracellular diffusion of endotoxin from intestinal lumen into circulation. The increase of endotoxin levels in GBF birds indicates that gut barrier failure increased the transport of endotoxin from intestinal lumen into circulation, which could further negatively affect the integrity of TJ as evidenced by the decrease of occludin mRNA levels in GBF birds. Occludin, one of the major components of TJ, is involved in the regulation of inter-membrane diffusion and paracellular diffusion of small molecules (64). Occludin is down-regulated in patients with Crohn’s Disease and ulcerative colitis, two common types of inflammatory bowel disease in humans (57, 64), suggesting the important role of occludin in intestinal health. However, no differences were detected between GBF and CON chickens in the expression of other TJ components, such as claudin 1, ZO1, JAM2, and JAM3. Claudin 1 is a member of multiple-span transmembrane protein called claudins, a protein family with more than 20 members, JAM2 and JAM3 are single-span transmembrane protein (51, 65, 66). ZO1 is a plaque protein that acts as adaptors to connect transmembrane proteins to the perijunctional actomyosin ring (23). These results indicate that GBF model impaired TJ integrity by reducing occludin expression, which facilitates the transport of endotoxin from intestinal lumen into blood for systemic circulation.

Endotoxin was also reported to increase satiety peptide secretion, which will reduce FI (20). The decreased growth performance in GBF birds could be partially associated with the increase of satiety peptide resulting from the elevated endotoxin levels, although satiety peptide was not measured in this study.

α1-Acid glycoprotein, an acute phase protein, has been used as a marker for systemic inflammation in poultry (67). Increase of AGP in GBF birds confirms that systemic inflammation was occurring in GBF birds, which led us to investigate the local inflammation status in the gut. Changes in the gut microbiota have been reported to negatively affect gut barrier integrity, leading to increased leakage of endotoxin and fatty acids, which can act upon TLR4 to activate systemic inflammation (68). Activation of macrophages via TLR is important for inflammation and host defense against pathogens; however, recent studies suggest that non-pathogenic molecules are able to induce inflammation via TLR2 and TLR4 (16, 69–71). The capacity to detect tissue injury and to initiate adequate repair mechanisms is indispensable for the survival of all higher species. A common aspect of all types of injury – caused by infectious, physical, chemical, or immune processes – is a compositional change of the cellular environment leading to the presence of novel molecular patterns. These patterns are recognized by a group of receptors termed pattern recognition receptors (PRR) and trigger specific responses that promote the restoration of tissue function, including inflammation and wound healing (20, 72). Pathogen recognition is critical to survive in an essentially hostile environment that is full of potentially infective microorganisms. Detection systems for molecular patterns characteristic for pathogens (pathogen-associated molecular patterns, PAMP) develop early in evolution, and are present in most species including plants and invertebrates (69). As a group of highly conserved PRR, TLR signals the presence of various PAMP to cellular constituents of the innate and adaptive immune (69, 73), therefore acting as gatekeepers for several highly efficient response systems that regulate tissue homeostasis and protect the host after acute injury (60, 74). Upon injury, the intestinal epithelium undergoes a wound healing process (69). Recent studies have revealed the activation of TLR by the microbiota during the healing process (20). In addition, several cytokines, such as TGF-α, TGF-β, IL-1β, and IL-2, are also increased during healing process (16, 75). In this study, the mRNA levels of TGF-β and IL-1β in GBF chickens were increased, but TLR4, TLR2β, TGF-α, IL-4, and IL-6 mRNA levels were not different compared to CON chickens. These results suggest that the inflammation occurred in GBF birds in this study is likely not mediated by TLR2 or TLR4 pathway. However, TLR3 mRNA and protein levels of TLR2, TLR4, and TLR3 were not measured in this study. Therefore, we are not able to exclude the possibility that TLR pathway is involved in the inflammation in GBF birds. IL-1β is an important mediator of the inflammatory response and is involved in a variety of cellular activities, including cell proliferation, differentiation, and apoptosis (76). TGF-β, a key mediator of mucosal immune homeostasis, mediates IgA production, retains lymphocytes in the gut and promotes wound healing of intestinal epithelium and mucosa (75). TGF-β also promotes IEC proliferation through the activation of extracellular signal-regulated kinase (ERK) 1/ERK2 mitogen-activated protein kinase during wound healing (20). IL-8 is secreted basolaterally by intestinal epithelium in response to pathogenic bacteria or specific inflammory cytokines, and triggers neutrophil migration and inflammation in intestine (73). The increase of systemic AGP, and mucosal TGF-β, IL-8, and IL-1β in GBF birds indicate that GBF model increased intestinal inflammation and activated intestinal innate immune response and wound healing.

Mucins are large glycoproteins that cover epithelial surfaces of the intestine and form a mucus layer to protect epithelial cells from gut health challenge. There are two major types of mucins, membrane-bound and secreted (77, 78). In chickens (*Gallus gallus*), three transmembrane mucins (MUC4, MUC13, and MUC16) and four gel-forming mucins (MUC6, MUC2, MUC5ac, and MUC5b) have been identified (79). In mammals, MUC2, the mucin secreted by goblet cells, is the most abundant mucin in the intestine, and its deficiency has been reported to increase bacterial translocation and inflammation (18, 80). Evolutionary studies suggest that mucins share a common ancestor, since their domain structures are well conserved in metazoans (71, 81). All mucins (MUC) contain at least one PTS domain, a region rich in proline, threonine, and serine (18, 82). Chicken MUC2 has been reported to be remarkably similar to human and mouse outside of the central PTS domain, but is highly divergent within this central repetitive structure (82, 83). Although the physiological implications and disease associations of MUC on various mucosal surfaces are well understood, there are still many questions as to how and why the gene architecture of this family contributes to diverse protein modifications that show diverse biological effects between metazoans in health and disease (18, 84–87). MUC2 gene expression has been used as a marker for gut health in poultry and other species (85, 88, 89). For example, Li et al. found that zinc supplementation in breeder diets improved morphometry, increased the number of goblet cell per villus, and MUC2 gene expression, and reduced mRNA levels of proinflammatory cytokines, such as IL-6 and IL-1β in the jejunum of their offspring (89). In the present study, MUC2 gene expression was reduced by 29% in GBF birds compared to CON birds, suggesting that GBF model reduced mucus layer protection in jejunum.

Intracellular lipid chaperones known as FABP are a group of molecules that coordinate lipid response and metabolism in cells (90). FABP are found across species, from *Drosophila melanogaster* and *Caenorhabditis elegans* to mice and humans, demonstrating strong evolutionary conservation (90). FABP-mediated lipid metabolism is closely linked to both metabolic and inflammatory processes through modulating critical lipid-sensitive pathways in target cells, especially adipocytes and macrophages (90, 91). Nine FABP have been identified so far in intestine, liver, brain, adipose, and muscle, the organs that show high rates of lipid metabolism, in vertebrates (92, 93). Intestinal FABP, FABP2, and FABP6, are expressed at high levels in the small intestine and ileum, respectively, and in addition to mediate lipid metabolism, they are also involved in intestinal inflammatory conditions by modulating critical lipid-sensitive pathways in adipocytes and macrophages in human (94, 95). FABP2 is down-regulated in patients with ischemia/reperfusion-induced intestinal barrier injury (93), suggesting the important role of FABP2 in gut barrier health. FABP2 has been identified as a specific marker for the relative amount of epithelium in humans and pigs (96). Several FABP (FABP1, FABP2, FABP6, and FABP10) have been identified to be predominantly expressed in the digestive tract of chickens (97, 98); however, much remains to be determined regarding their expression and biological functions in poultry. FABP10 plays an important hepatic role in in response to FI in chicken (98). FABP2 is involved in lipogenesis and fatty acids transport, and plays an important role in abdominal fat content in broiler chickens (98–100). In the present study, GBF model reduced FABP2 gene expression, suggesting that, like the role of FABP2 in human intestinal barrier health, FABP2 can be used as a marker of gut barrier function in chicken. Reduction of FABP2 expression indicates the loss of epithelial cell content and occurrence of intestinal barrier failure in GBF birds.

The ileal lipid binding protein (ILBP; human gene FABP6) was recently shown to be needed for the efficient transport of bile acids from the apical side to the basolateral side of enterocytes in the distal portion of murine intestine (101). Bile acids are synthesized by the liver and released into the lumen of the small intestine via bile, and the majority of bile acids are recovered in the distal end of the small intestine and then returned to the liver for reuse (102). Bile acid has emerged as important biological molecules that emulsify lipids and liposoluble dietary nutrients to facilitate their digestion and absorption (103, 104). It has strong anti-microbial activity and therefore is emerging as a host factor that regulates the composition of microbiota in the gut (105, 106). Reduced bile acid levels in the gut are reported to be associated with bacterial overgrowth and inflammation (106). Gut inflammation in GBF birds may have resulted in lower levels of bile acids, which unfortunately were not measured in this study. The substantial increase of FABP6 by four fold in GBF birds indicates high demand of bile acids as an anti-microbial to promote the recovery of dysbacteriosis and barrier failure in the gut of GBF birds.

In conclusion, the purpose of this study was not to determine the individual effects of diet ingredients or coccidia challenge but rather to determine the potential biomarkers that may be used to define gut barrier failure in future studies. We attempted to exacerbate gut barrier failure with the tools available for us, and the results obtained in the present study suggest that the combination of high NSP diet and a coccidia challenge induced gut barrier failure and inflammation in broilers characterized by the increase of endotoxin and AGP in serum, as well as increase of IL-8, IL-1β, TGF-β4, and FABP6 mRNA, and reduction of FABP2, MUC2, and occludin mRNA in jejunal mucosa of GBF birds compared to CON birds. These parameters may be utilized as potential biomarkers for gut barrier health in chickens. Now that we have a better understanding of what biomarkers are relevant in gut barrier failure models in chickens, further studies will be conducted to evaluate the effects of chicken enteropathogens, different dietary ingredients or feed additives, such as probiotics and prebiotics, on gut barrier function in broiler chickens.

## Conflict of Interest Statement

The authors declare that the research was conducted with financial support of Novus International Inc. Mention of trade names or commercial products in this article is solely for the purpose of providing specific information and does not imply recommendations or endorsement by Novus International Inc.

## Abbreviations

AGP, α1-acid glycoprotein; AJ, adherens junctions; BW, body weight; BWG, body weight gain; CON, control; ERK, extracellular signal-regulated kinase; FABP, fatty acid-binding protein; FCR, feed conversion ratio; FI, feed intake; GBF, gut barrier failure; GIT, gastrointestinal tract; IEC, intestinal epithelial cells; IL, interleukin; JAM, junctional adhesion molecule; MUC2, mucin 2; NSP, non-starch polysaccharide; PAMP, pathogen-associated molecular pattern; PRR, pattern recognition receptor; TGF, transforming growth factor; TJ, tight junction; TLR, toll-like receptor; TNF, tumor necrosis factor; ZO, zonula occluden.
